# Meaningful use: a roadmap for the advancement of health information exchange

**DOI:** 10.1186/2045-4015-2-26

**Published:** 2013-07-23

**Authors:** Michael F Furukawa

**Affiliations:** 1 Department of Health and Human Services, Office of Economic Analysis, Evaluation and Modeling, Office of the National Coordinator for Health Information Technology, 200 Independence Ave SW, Washington, DC 20201, USA

## Abstract

Frankel and colleagues have compared Israel and the U.S.’s experiences with health information exchange (HIE). They highlight the importance of institutional factors in fostering HIE development, notably the influence of local structures, experience and incentives. Historically, information infrastructure in the U.S. has been limited due to lack of standards, fragmented institutions and competition. The Health Information Technology for Economic and Clinical Health (HITECH) Act of 2009 authorized billions of dollars for the adoption and “Meaningful Use” of electronic health records. HITECH programs and Meaningful Use incentives target the advancement of HIE through 1) building blocks, 2) local support and 3) payment incentives. Meaningful Use requirements create a roadmap to broader electronic exchange of health information among providers and with patients. Ultimately, successful HIE in the U.S. will depend on whether Meaningful Use can address institutional needs within local markets.

This is a commentary on http://www.ijhpr.org/content/2/1/722

## Commentary

Health information technology (health IT) has the potential to improve the safety, efficiency and quality of patient care [1]. Health information exchange (HIE) can enhance patient care by allowing information to follow patients as they transition across providers and settings [2]. HIE can also engage consumers through access to their health information allowing patients to switch providers and members to switch health plans [3]. National policies in the U.S. and abroad have focused on the adoption of health IT with the goals of widespread sharing of clinical information among providers and with patients [4].

In Frankel and colleagues’ article published recently in *The Israel Journal of Health Policy Research* the authors analyze the role of institutional factors in the development of HIE initiatives in Israel and the U.S. [5]. In both countries, the evolution of HIE entities has been shaped by local structures, experience and incentives. In Israel, a coordinated HIE strategy emanates from national health insurance administered by four Health Maintenance Organizations (HMOs). Information sharing is facilitated by experience with a national electronic medical registry and some vertical integration across hospitals and community providers.

In the U.S., HIE strategy has evolved over time within a competitive, fee-for-service environment with fragmented providers. Early policies focused on development of a nationwide health information network, an architecture that sought to connect community-level HIE entities within regional markets [6]. Regional health information organizations (RHIOs) have emerged in states and local communities across the U.S. In 2012, 119 operational HIOs connected 30% of hospitals and 10% of providers [7]. Despite modest growth of HIE entities, electronic exchange activity outside organizational boundaries has been limited by competitive pressures and interoperability across vendors [8,9].

## Meaningful Use created a roadmap to advance health information exchange

In 2009, the national strategy for the advancement of HIE shifted with the Health Information for Economic and Clinical Health (HITECH) Act of 2009. HITECH authorized the Office of the National Coordinator for Health Information Technology to implement programs and created the Medicare and Medicaid Electronic Health Records Incentive Programs to award incentive payments to physicians and hospitals to adopt and meaningfully use interoperable electronic health records (EHRs) [10,11]. HITECH programs and Meaningful Use incentives target the advancement of HIE through the development of building blocks for interoperability, provision of local support for infrastructure and payment incentives to share information outside the organization [12]. In terms of the terminology used by Frankel and colleagues, these are key institutional considerations for the design and implementation of HIE.

## Standards and certification are building blocks for interoperability

Widespread information sharing requires the technical ability to securely and reliably send and receive structured data from disparate sources. In the U.S., the national HIE strategy focuses on standards, policies and services as building blocks to enable the exchange of health information through regional HIOs and/or through interoperable EHRs [12]. The Standards and Interoperability Framework focuses on voluntary, consensus-based standards to enable the key forms of exchange (directed, query-based, consumer-mediated). Policies are focused on HIE governance, ensuring privacy and security, and reducing the cost and complexity of exchange. The Health IT Certification Program ensures that certified EHR technology can meet the HIE requirements for Meaningful Use.

## Local support for infrastructure can address barriers to exchange

Starting conditions and institutional context may vary across communities resulting in unique barriers to HIE. For example, a key barrier to sharing health information is limited existing infrastructure to connect unaffiliated providers and hospitals. The provision of technical assistance can aid stakeholders in overcoming these barriers. HITECH created three programs at the local-level to develop infrastructure and assist with Meaningful Use [11]. The State Health Information Exchange Cooperative Program funded 56 state designated entities to enact policies and offer services to enable HIE within each state and territory. The Regional Extension Center (REC) program funded 62 local organizations to assist primary care providers in small practices and underserved settings in meeting Meaningful Use requirements. The Beacon Community program funded 17 communities to demonstrate the benefits of health IT, including building and strengthening HIE. These HITECH programs may be a catalyst for local change and further investment, and some states have expanded upon HITECH with further investments in infrastructure including HIE support [13].

## Payment incentives create a business case for information sharing

Beyond building blocks and local support, national policies in the U.S. have focused on aligning Meaningful Use with payment incentives to create a business case for information sharing outside the organization. Meaningful Use incentives require providers and hospitals to exchange health information with outside providers and with patients through certified EHR technology. Stage 2 requirements include the exchange of transitions of care summaries and the capability to share standards-based data across vendor platforms [14]. Beyond Meaningful Use incentives, Medicare payment policies have embedded health IT into requirements for electronic prescribing and quality reporting through the Centers for Medicare & Medicaid Services [15]. Further alignment of health IT and payment incentives is evidenced by the embedding of Meaningful Use requirements in public and private accreditations for patient-centered medical home and accountable care organization initiatives [16].

Institutional factors highlighted by Frankel and colleagues will continue to shape the context and incentives for broader electronic exchange of health information. In the U.S., the national strategy leverages Meaningful Use as a roadmap to advance HIE through regional HIOs and interoperable EHRs. Policies and programs address institutional challenges through building blocks, local support and payment incentives. Standards and certification enable providers, hospitals, and patients to share health information through interoperable EHRs. State designated entities and regional extension centers provide technical assistance to meet the Meaningful Use requirements for electronic exchange. Finally, the alignment of Meaningful Use with payment incentives creates a business case for information sharing outside the organization. Ultimately, the future success of HIE in transforming patient care and empowering consumers in the U.S. will depend on whether Meaningful Use can address the needs of institutions within local markets.

## Competing interests

The author declares that they have no competing interests.

## Author information

Michael F. Furukawa is the Director of the Office of Economic Analysis, Evaluation and Modeling at the Office of the National Coordinator for Health Information Technology (ONC) in the U.S. Department of Health and Human Services in Washington D.C. Previously, he was an Assistant Professor in Health Management and Policy at Arizona State University.

## Commentary on

Frankel M, Chinitz D, Salzberg CA, Reichman. Sustainable Health Information Exchange: The Role of Institutional Factors. The Israel J of Health Policy Research 2013, 2:21.
